# The analgesic effect of refeeding on acute and chronic inflammatory pain

**DOI:** 10.1038/s41598-019-53149-7

**Published:** 2019-11-14

**Authors:** Jeong-Yun Lee, Grace J. Lee, Pa Reum Lee, Chan Hee Won, Doyun Kim, Youngnam Kang, Seog Bae Oh

**Affiliations:** 1 Department of Brain and Cognitive Sciences, College of Natural Sciences, Seoul National University, Seoul, Republic of Korea; 2 Dental Research Institute and Department of Neurobiology & Physiology, School of Dentistry, Seoul National University, Seoul, Republic of Korea; 3 Department of Behavioral Physiology, Graduate School of Human Sciences, Osaka University, Osaka, Japan

**Keywords:** Preclinical research, Pain management

## Abstract

Pain is susceptible to various cognitive factors. Suppression of pain by hunger is well known, but the effect of food intake after fasting (i.e. refeeding) on pain remains unknown. In the present study, we examined whether inflammatory pain behavior is affected by 24 h fasting and 2 h refeeding. In formalin-induced acute inflammatory pain model, fasting suppressed pain behavior only in the second phase and the analgesic effect was also observed after refeeding. Furthermore, in Complete Freund’s adjuvant-induced chronic inflammatory pain model, both fasting and refeeding reduced spontaneous pain response. Refeeding with non-calorie agar produced an analgesic effect. Besides, intraperitoneal (i.p.) administration of glucose after fasting, which mimics calorie recovery following refeeding, induced analgesic effect. Administration of opioid receptor antagonist (naloxone, i.p.) and cannabinoid receptor antagonist (SR 141716, i.p.) reversed fasting-induced analgesia, but did not affect refeeding-induced analgesia in acute inflammatory pain model. Taken together, our results show that refeeding produce analgesia in inflammatory pain condition, which is associated with eating behavior and calorie recovery effect.

## Introduction

Pain perception is a multifaceted experience, largely divided into sensory and affective dimension^1,2^. The sensory dimension of pain provides sensory-discriminative information such as location, quality, and intensity. Affective pain consists of hedonic aspect (pleasantness/unpleasantness) and affective-motivation aspect which creates behavior to escape from pain as a secondary pain effect^3^. Since pain is both a sensation and the emotion which is an unpleasant state motivating an organism to react in favor of its survival, it is susceptible to modulation by cognitive factors^4,5^.

Hunger is well known as a powerful driving force to change cognition. Several clinical evidences have shown that mood states, such as confusion, anger, and tension can be improved by limiting food intake^6^. Furthermore, therapeutic fasting is also effective for pain relief and mood enhancement regardless of body weight change in the chronic pain patient^7,8^. In various animal pain models, it is also well established that fasting or calorie restriction also has an analgesic effect^9–12^. Interestingly, recent studies have revealed that a part of the parabrachial nucleus (PBN) is involved in the suppression of pain response by fasting^13,14^. Endogenous opioid and endocannabinoid system not only play a critical role in both homeostatic and hedonic aspects of feeding but also are involved in the endogenous pain inhibitory system^15,16^. Therefore, activation of opioid and endocannabinoid system after fasting is likely to modulate pain. However, the relationship between the analgesic effect after fasting and these endogenous pain control systems have not been elucidated.

While several studies have found the fasting-induced analgesia, little is known about the effect of food intake after fasting on pain. Our previous study has revealed that hedonic drinking induces an analgesic effect in fasted rats^17^. Moreover, food ingestion after fasting reduced pain perception in healthy volunteers^18^. In the animal study, thermal pain threshold also increased at returning to free feeding after calorie-restriction^19^. Thus these studies strongly suggest the possibility that food intake after fasting can suppress pain. Nevertheless, the phenomenon or the mechanism for it is still unknown.

In this study, we thus sought to explore the change of pain behavior following fasting and refeeding using two inflammatory pain conditions with the formalin-induced acute pain model and Complete Freund’s adjuvant (CFA)-induced chronic pain model. We found that both fasting and refeeding produce an analgesic effect on inflammatory pain and refeeding-induced analgesia is mediated by eating behavior and calorie recovery. The opioid and endocannabinoid system is only associated with fasting-induced analgesia, but not with refeeding-induced analgesia.

## Results

### Both fasting and refeeding suppress acute inflammatory pain behavior

**Figure 1:**
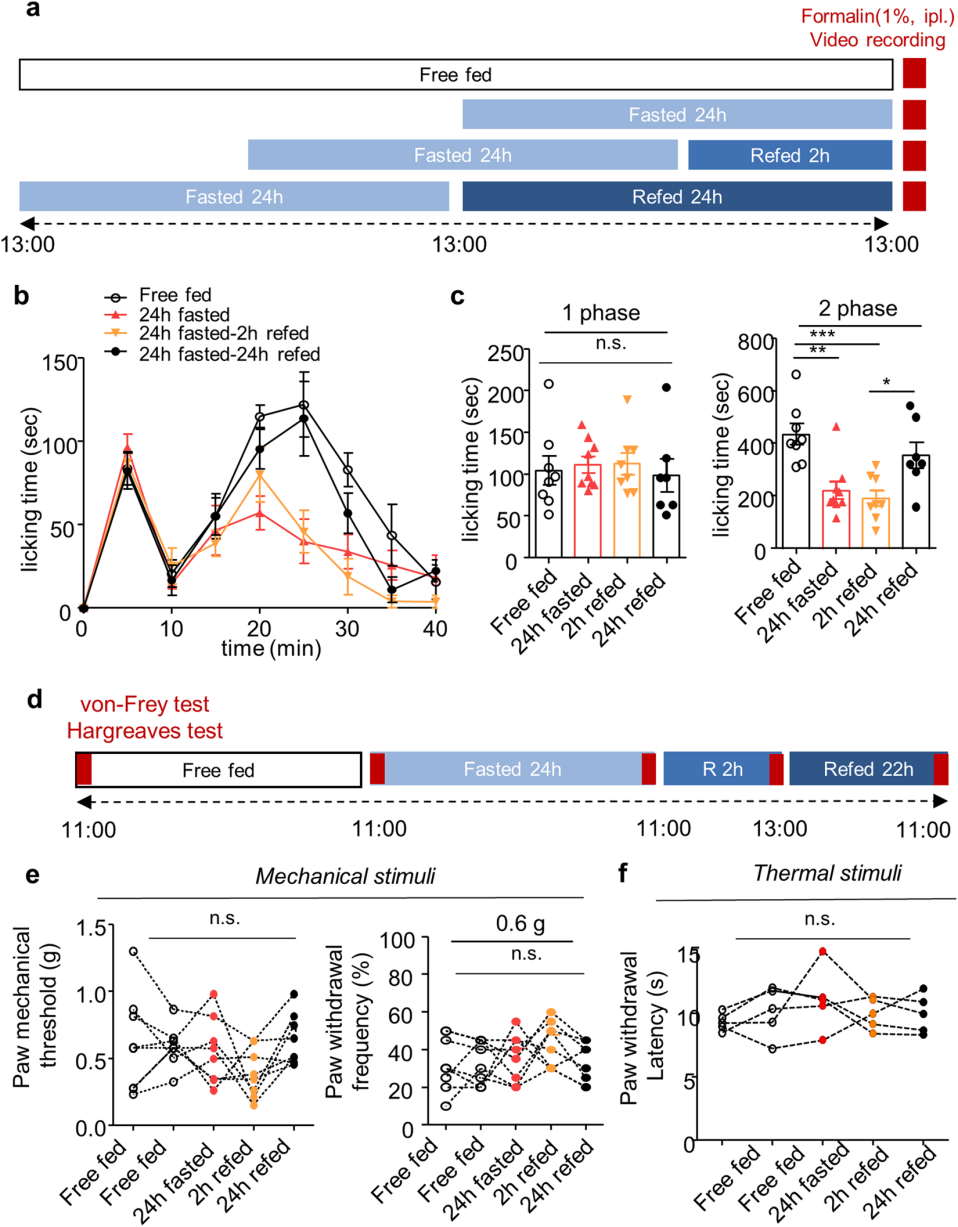
The effect of fasting and refeeding on acute pain behavior. (**a)** Experimental design and schedule for formalin test. (**b)** Time course of spontaneous pain behavior following intraplantar injection of formalin. (**c)** Formalin-induced pain behavior was divided into two phases and the total sum of the licking times for each phase was statistically analyzed; Free fed (n = 8), 24 h fasted (n = 9), 2 h refed (n = 8), 24 h refed (n = 7). (**d)** Experimental design and schedule for von Frey test and Hargreaves test on naïve mice; (n = 6). (**e,f)** The effect of 24 h fasting and 2 h refeeding on paw withdrawal threshold and frequency for mechanical stimuli (n = 6) and paw withdrawal latency for thermal stimuli (n = 5). Data are presented as mean ± SEM. *p < 0.05, **p < 0.01, ***p < 0.001 (**c** one-way ANOVA followed by Bonferroni test, **e, f** repeated measures ANOVA).

To investigate the effect of fasting and refeeding on acute pain behavior, we measured spontaneous pain behavior in the formalin-induced acute inflammatory pain model and mechanical/thermal pain threshold in naïve mice (Fig. 1). To make satiety condition, mice were fasted for 24 h and then allowed to free access to normal chow for 2 h. Induction of satiety was confirmed by body weight, blood glucose level, food intake and stomach size (Supplementary Fig. S1). After 24 h fasting, body weight and blood glucose level decreased, which was recovered after 2 h refeeding (Supplementary Fig. S1a,b). 2 h refeeding increased food intake and stomach was significantly expanded compared to the free fed mice (Supplementary Fig. S1c,d)

Consistent with previous studies^11,14,20^, our results also showed that 24 h acute fasting suppressed formalin-induced spontaneous pain behavior with a significant analgesic effect only in the second phase (Fig. 1a–c). As compared with the free fed group, 2 h refed group had a significant analgesic effect only in the second phase, and this analgesic effect was disappeared at 24 h refeeding (Fig. 1a–c). In naïve mice, both paw withdrawal threshold for mechanical stimuli and paw withdrawal latency for thermal stimuli were not affected by 24 h fasting and remained unchanged after 2 h/24 h refeeding (Fig. 1d–f).

These results suggest that both fasting and refeeding produces an analgesic effect in acute inflammatory pain but dose not affect non-inflammatory pain (nociception) (Table 1).

**Table 1:** The effect of fasting and refeeding on different types of pain.

			Acute pain		Chronic pain	
			24 h Fasted	2 h Refed	24 h Fasted	2 h Refed
Inflammatory pain	*Spontaneous pain*		Analgesia	Analgesia	Analgesia	Analgesia
	*Evoked* *pain*	*Mechanical allodynia*	—	—	No effect	Analgesia
		*Thermal hyperalgesia*	—	—	No effect	No effect
Non-Inflammatory Pain (nociception)	*Chemical stimuli*		No effect	No effect	—	—
	*Mechanical stimuli*		No effect	No effect	—	—
	*Thermal stimuli*		No effect	No effect	—	—

The type of pain was divided according to etiologic classification: *inflammatory / non-inflammatory pain (nociception)* and symptomatic classification: *spontaneous pain / evoked pain*^45,46^. Acute inflammatory pain was confirmed in 2^nd^ phase of the formalin test. Acute non-inflammatory pain (nociception) was confirmed in 1^st^ phase of the formalin test, von Frey test and Hargreaves test. In the chronic inflammatory model, spontaneous pain response was confirmed by complete Freund’s adjuvant (CFA)-induced licking and flinching behavior and mechanical allodynia was confirmed by von Frey test and thermal hyperalgesia was confirmed by Hargreaves test.

### Fasting and refeeding produce differential analgesic effects in chronic inflammatory pain model

**Figure 2:**
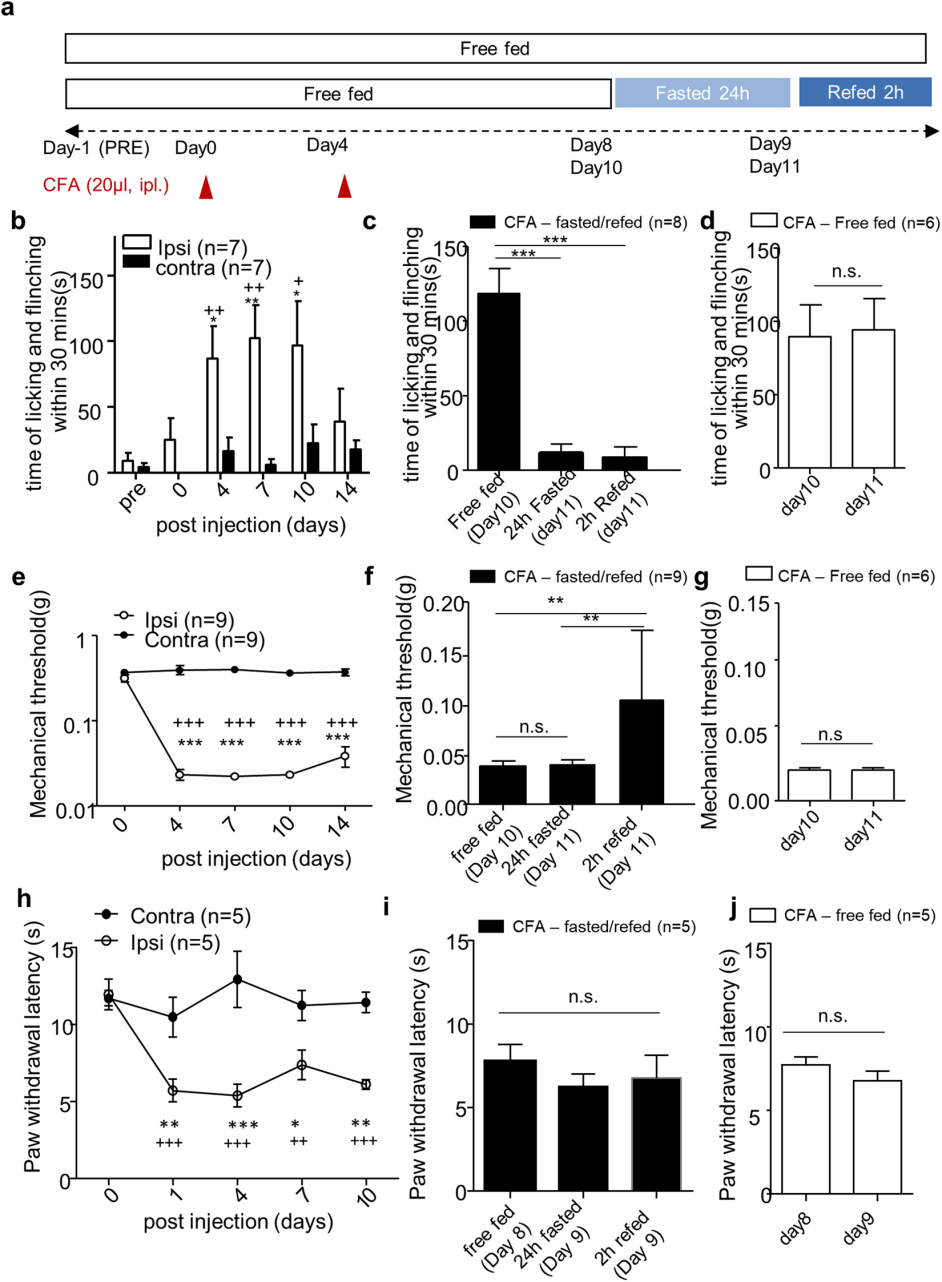
The effect of fasting and refeeding on chronic inflammatory pain behavior. (**a**) Experimental design and schedule for complete Freund’s adjuvant (CFA)-induced chronic inflammatory pain model. (**b)** Time course of spontaneous pain behavior following CFA injection; (n = 7). (**c,d)** The effect of 24 h fasting and 2 h refeeding on CFA-induced spontaneous pain behavior; Free fed (n = 6), Fasted/Refed (n = 8). (**e)** Time course of mechanical allodynia following CFA injection; (n = 9). (**f,g)** The effect of 24 h fasting and 2 h refeeding on CFA-induced mechanical allodynia; Free fed (n = 6), Fasted/Refed (n = 9). (**h)** Time course of thermal hyperalgesia following CFA injection; (n = 5). (**i,j)** The effect of 24 h fasting and 2 h refeeding on CFA-induced thermal hyperalgesia; Free fed (n = 5), Fasted/Refed (n = 5). Cross denote significance levels in comparison with pre-injection. Artistes denote significance levels in comparison with a contralateral hind paw. Data are presented as mean ± SEM. *p < 0.05, **p < 0.01, ***p < 0.001, +p < 0.05, ++p < 0.01, ++p < 0.001 (**b,e,h** two-way ANOVA followed by Bonferroni, (**c,f,i)** repeated measures ANOVA followed by Bonferroni, (**d,g,j** paired t-test, two-tailed).

We next examined the effect of fasting and refeeding on chronic inflammatory pain. Given spontaneous pain behavior does not last more than 7 days in the conventional CFA model^21^, we generated a new model to observe spontaneous pain behavior in chronic inflammatory pain model. When the mice received a booster injection of CFA 4 days after the first injection (Fig. 2a), we found that CFA-induced spontaneous pain behavior significantly increased from day 4 to day 11, compared to pre-injection and contralateral hind paw, and this spontaneous pain behavior started to decline after day 14 (Fig. 2b). CFA-induced mechanical allodynia lasted for 14 days in the von Frey test (Fig. 2e) and CFA-induced thermal hyperalgesia lasted for 10 days in Hargreaves test (Fig. 2h).

When food was removed on day 10 in the CFA-induced chronic pain model, body weight and blood glucose level on day 11 after 24 h fasting was decreased and then recovered after 2 h refeeding, like in naïve mice (Supplementary Fig. S2a,b). However, the body weight decreased and the food intake was not constant while pain persisted, as compared to naïve mice (Supplementary Fig. S2d,e). When we compared pain behavior on day 11 to day 10, CFA-induced spontaneous pain behavior was significantly reduced by 24 h fasting and 2 h refeeding (Fig. 2c), while the free fed group had no change in pain behavior (Fig. 2d). On the other hand, mechanical allodynia in von Frey test decreased only in 2 h refeeding, but not in 24 h fasting (Fig. 2f). Again, the free fed group had no change in mechanical allodynia behavior (Fig. 2g). Thermal hyperalgesia in Hargreaves test was not affected by 24 h fasting and 2 h refeeding (Fig. 2i,j).

Collectively, our results showed that fasting alleviated only spontaneous pain behavior but not mechanical allodynia/thermal hyperalgesia. However, refeeding reduced both spontaneous pain behavior and mechanical allodynia in the chronic inflammatory pain model (Table 1).

### Refeeding of non-calorie agar pellet induces analgesic effect

Next, we investigated whether eating behavior or calorie recovery is involved in refeeding-induced analgesia. To eliminate the effect of calorie recovery, we used non-calorie agar pellet.

**Figure 3:**
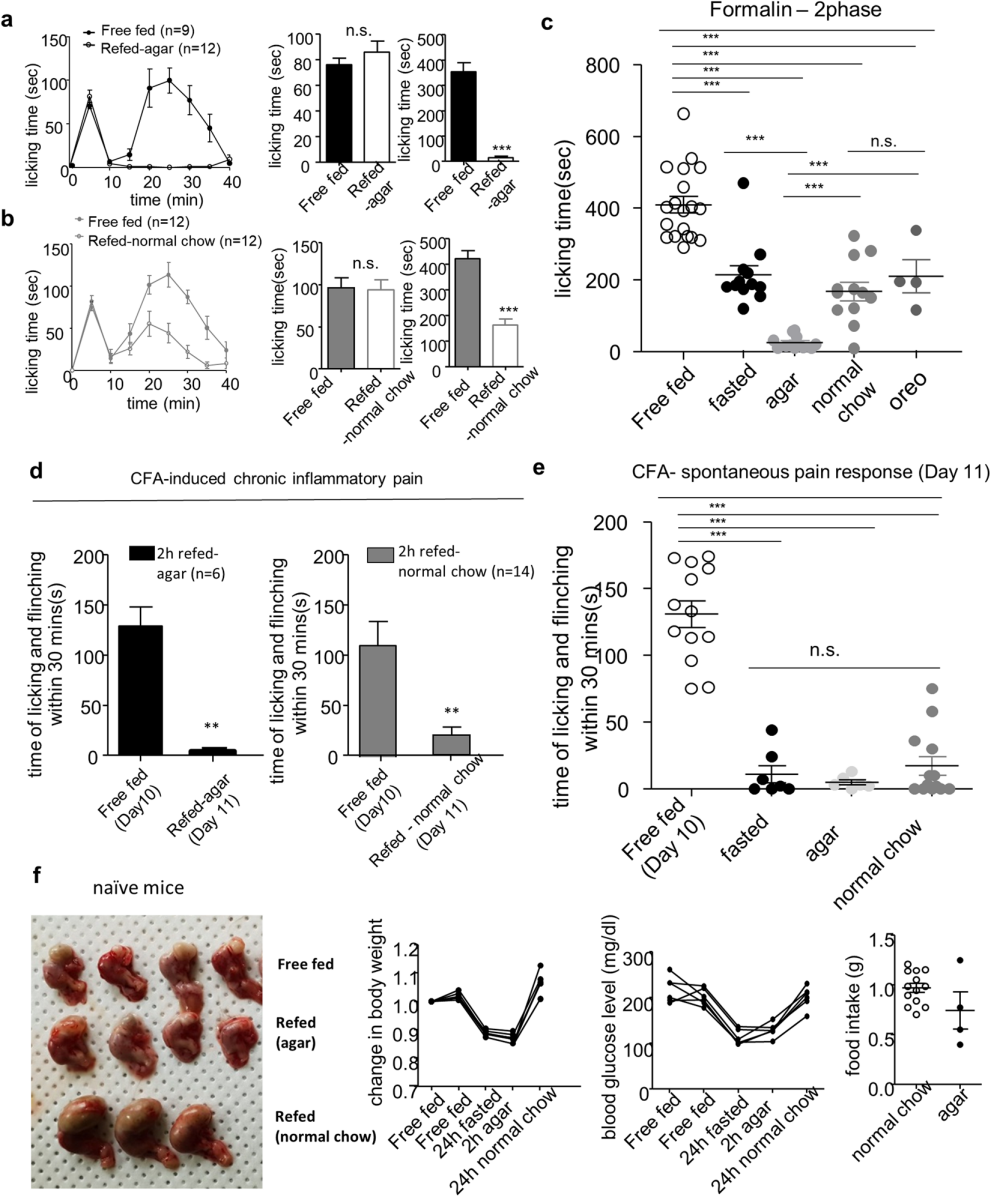
The analgesic effect of refeeding with non-calorie agar on inflammatory pain behavior. (**a,b)** Effect of 2 h refeeding non-calorie agar or normal chow on formalin-induced pain behavior; Free fed (n = 9), Agar-refed (n = 12), Free fed (n = 12), Normal chow-refed (n = 12). (**c)** Comparison of 2^nd^ phase according to experimental groups; Free fed (n = 19), Fasted (n = 12), Agar-refed (n = 12), Normal chow-refed (n = 12), Oreo-refed (n = 4). (**d)** Effect of 2 h refeeding non-calorie agar or normal chow on CFA-induced spontaneous pain behavior; Agar-refed (n = 6), normal chow-refed (n = 14). (**e)** Comparison of CFA-induced spontaneous pain behavior on day 11 according to experimental groups; Free fed (n = 13), Fasted (n = 7), Agar-refed (n = 6), Normal chow-refed (n = 14). (**f)** Change in stomach size, body weight, blood glucose level and food intake after agar refeeding in naïve mice. Data are presented as mean ± SEM. **p < 0.01, ***p < 0.001 (**a,b** unpaired t-test, two-tailed, **c,e** one-way ANOVA followed by Bonferroni test, **d** paired t-test, two-tailed).

In the formalin-induced acute pain model, both refeeding of non-calorie agar and normal chow had an analgesic effect only in the second phase (Fig. 3a,b). Interestingly, the agar-refeeding group had a greater analgesic effect than the fasting or normal chow-refeeding group (Fig. 3c), whereas there was no difference in the analgesic effect between high-calorie chow (Oreo) and normal chow (Fig. 3c). In the CFA-induced chronic inflammatory pain model, both agar and normal chow refeeding had an analgesic effect, but there was no difference between the two groups (Fig. 3d,e). As compared to refeeding with normal chow, refeeding of non-calorie agar for 2 h did not increase stomach size, body weight, and blood glucose level (Fig. 3f). Thus, agar-refeeding engaged eating behavior but did not induce satiety signal by calorie recovery or stomach expansion.

Our results indicate that eating behavior during refeeding contributes to refeeding-induced analgesia, regardless of calorie.

### Intraperitoneal administration of glucose induces analgesic effect

An additional analgesic effect by refeeding with non-calorie agar suggests that satiety signal that consists of calorie recovery and stomach expansion may have interfered with refeeding-induced analgesia. So we next investigated the effect of calorie recovery on refeeding-induced analgesia. To determine the effect of calorie recovery on pain without engaging eating behavior and stomach expansion, we injected D-glucose (1 g/kg, i.p.).

**Figure 4:**
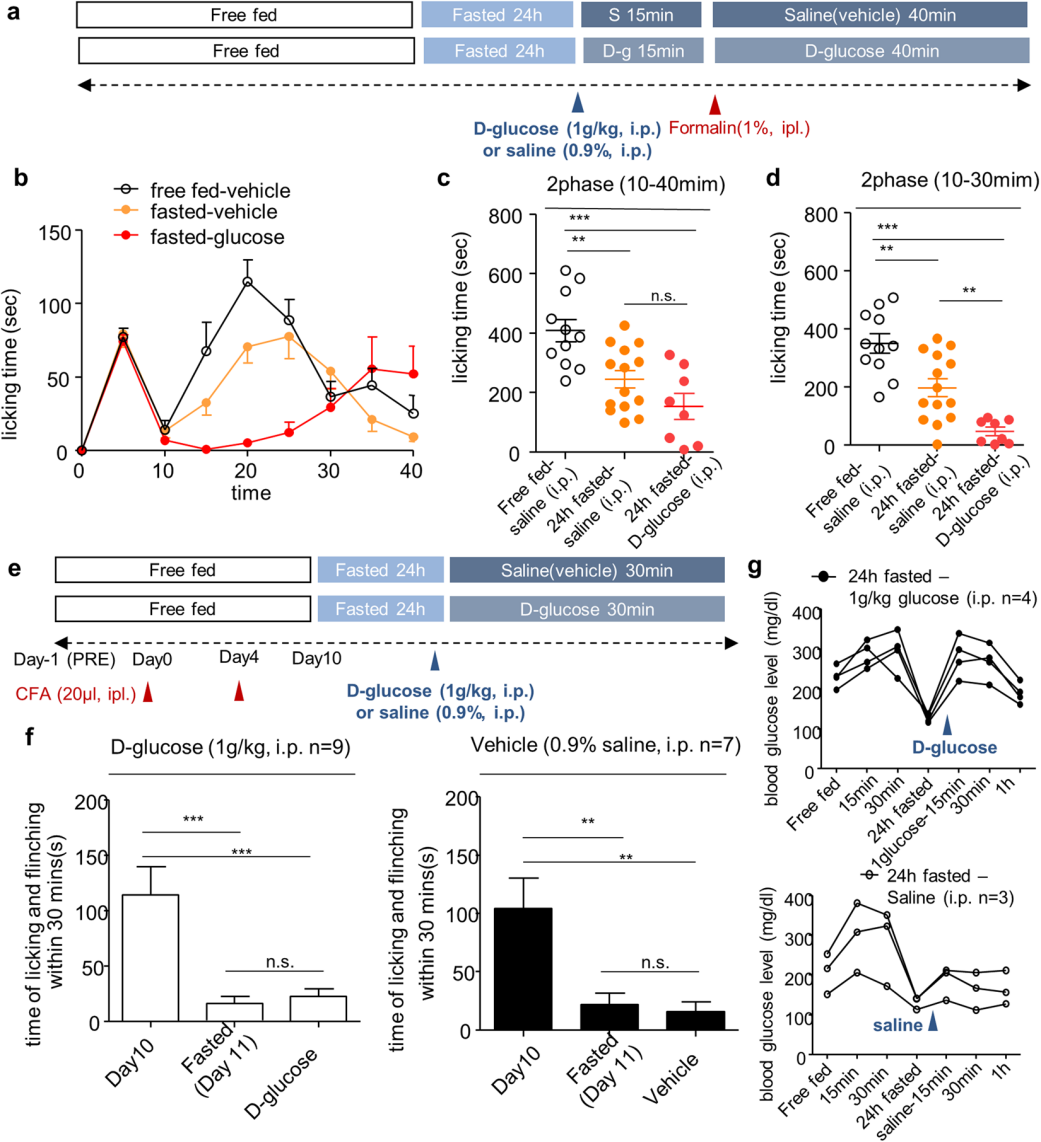
The analgesic effect of intraperitoneal administration of D-glucose on inflammatory pain behavior. (**a)** Experimental design and schedule for formalin test. (**b)** Time course of spontaneous pain behavior following intraplantar injection of formalin after D-glucose (1 g/kg) administration; Free fed (n = 11), Fasted (n = 14), D-glucose treated (n = 8). (**c,d)** 2^nd^ phase of the formalin test was divided into 10 to 40 min and 10 to 30 min and analyzed. (**e)** Experimental design and schedule for complete Freund’s adjuvant (CFA)-induced chronic inflammatory pain model; D-glucose treated (n = 9), Vehicle treated (n = 7). (**f)** Effect of D-glucose administration on CFA-induced spontaneous pain behavior. (**g)** Blood glucose level after intraperitoneal administration of D-glucose (1 g/kg); D-glucose treated (n = 4), Vehicle treated (n = 3). Data are presented as mean ± SEM. **p < 0.01, ***p < 0.001 (**c,d** one-way ANOVA followed by Bonferroni test, **f** repeated measures ANOVA followed by Bonferroni).

Formalin was injected 15 min after D-glucose injection in fasted mice (Fig. 4a). Compared with the vehicle (0.9% saline, i.p.) treated group, D-glucose treated group had no significant difference in the sum of formalin-induced spontaneous pain behavior from 10 min to 40 min (Fig. 4b,c). However, only when comparing formalin-induced pain behavior from 10 min to 30 min, pain behavior was significantly decreased in D-glucose treated group (Fig. 4b,d). In the CFA-induced chronic inflammatory pain model, the analgesic effect by the treatment of D-glucose was comparable to the analgesic effect by the vehicle injection (Fig. 4e,f). As compared to the vehicle group, the administration of D-glucose increased blood glucose level over time, peaked after 15 minutes and recovered after 1 h (Fig. 4g).

Taken together, our findings suggest that calorie recovery after fasting may serve as an additional factor for refeeding-induced analgesic effect.

### Opioid and endocannabinoid system contribute to fasting-induced analgesia, but not refeeding-induced analgesia

**Figure 5:**
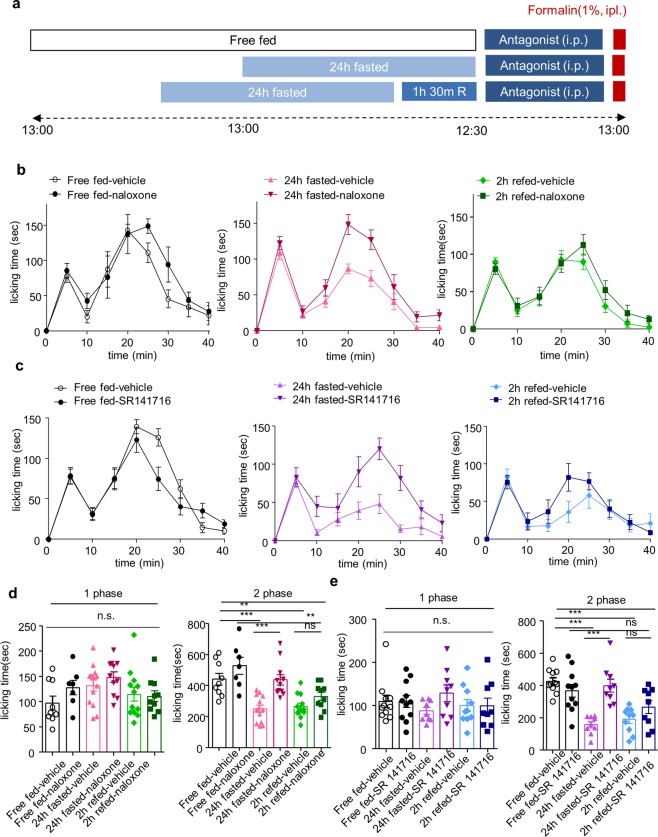
The effect of opioid receptor and cannabinoid receptor (CB1) antagonist on fasting and refeeding-induced analgesia. **(a)** Experimental design and schedule for formalin test. Naloxone (opioid receptor antagonist, 10 mg/kg) and SR 141716 (CB1 receptor antagonist, 10 mg/kg) were intraperitoneally (i.p.) administered 30 min before the formalin injection. (**b,c**) Time course of spontaneous pain behavior following intraplantar injection of formalin; Free fed-vehicle (n = 9), Free fed-naloxone (n = 7), 24 h fasted-vehicle (n = 13), 24 h fasted-naloxone (n = 11), 2 h refed-vehicle (n = 12), 2 h refed-naloxone (n = 10) / Free fed-vehicle (n = 11), Free fed-SR 141716 (n = 11), 24 h fasted-vehicle (n = 9), 24 h fasted-SR 141716 (n = 9), 2 h refed-vehicle (n = 10), 2 h refed-SR 141716 (n = 9). (**d,e)** Formalin-induced pain behavior was divided into two phase and the total sum of the licking times for each phase was statistically analyzed. Data are presented as mean ± SEM. **p < 0.01, ***p < 0.001 (**d,e** one-way ANOVA followed by Bonferroni test).

To determine the involvement of opioid and endocannabinoids system in fasting and refeeding-induced analgesia, we used naloxone (opioid receptor antagonist) and SR 141716 (cannabinoid receptor (CB1) antagonist). In the previous study, it was confirmed that SR 141716 significantly inhibit food intake after fasting at dose of 10 mg/kg but not at 3 mg/kg^22,23^. We also confirmed that 3 mg/kg of naloxone (i.p.) had no effect on fasting-induced analgesia (Supplementary Fig. 3). Naloxone (10 mg/kg, i.p.) and SR 141716 (10 mg/kg, i.p.) was administrated 30 min before formalin injection (Fig. 5a). In formalin-induced acute inflammatory pain model, both naloxone and SR 141716 inhibited fasting-induced analgesia but did not affect refeeding-induced analgesia (Fig. 5b–e).

Our results suggest that endogenous opioid and endocannabinoid system mediate fasting-induced analgesia, but not refeeding-induced analgesia. Refeeding might recruit distinctive analgesic factors from fasting-induced analgesia.

## Discussion

In the present study, we compared pain behaviors between fasting and refeeding mice and discovered that refeeding only alleviates pathological pain induced by inflammation. In the formalin-induced acute inflammatory pain model, refeeding suppressed only spontaneous pain behavior especially in the second phase which represents inflammatory pain. We confirmed that fasting and refeeding produces analgesic effects through different mechanisms where fasting produces analgesic effect via the opioid and endocannabinoid system, but these systems are not involved in refeeding-induced analgesia. Both refeeding of non-calorie agar and the calorie recovery by D-glucose injection (i.p.) after fasting had an additional analgesic effect, compared to fasting-induced analgesia. In CFA-induced chronic inflammatory pain model (twice CFA injection), refeeding reduced both spontaneous pain behavior and mechanical allodynia, whereas fasting only reduced spontaneous pain behavior.

Feeding behavior is crucial for maintaining homeostasis and it is well investigated that pain perception is changed in eating disorder patients^24,25^. Since opioid and endocannabinoid system are critical modulators of pain as well as feeding^15,16^, these systems are important factors in determining the relationship between feeding and pain. Diurnal fluctuations in pain sensitivity were not caused by circadian rhythm but food deprivation, which was related to the opioid system^9^. Intermittent fasting produced an analgesic effect via kappa-opioid system in the spinal cord^26^. It is also well known that leptin-deficient (*ob/ob*) obese mice have increased pain threshold by activating endocannabinoid system in descending pain pathway^27^. In the present study, we found that acute fasting leads to analgesic effects in acute inflammatory pain through opioid and cannabinoid system, whereas the analgesic effect of refeeding was not associated with these systems (Fig. 5). Thus refeeding might recruit distinctive factors which are different from fasting-induced analgesia.

Eating behavior and satiety signal such as calorie recovery and stomach expansion may result from refeeding after fasting. We determined which factors play critical roles in refeeding-induced analgesia. First, we identified the effect of eating behavior on pain. Several pre-clinical studies have suggested that pain signals are suppressed during drinking or eating regardless of calorie^17,28,29^. Moreover, our previous study also showed that not only drinking a sucrose solution but also water drinking increased pain thresholds in the Hargreaves test of thermal sensitivity^17^. However, these studies are not suitable to investigate the effects of refeeding on pain because eating and pain signals exist at the same time. In the present study, we performed a pain behavior test after eating behavior was completed, and found that even eating non-calorie food produces a greater analgesic effect than fasting-induced analgesia, indicating that eating behavior itself can suppress pain perception, regardless of calorie (Fig. 3).

Next, we examined the effect of calorie recovery after fasting on pain. Glucose administration after fasting is known to enhance neuroplasticity and cognitive function^30,31^, so it is possible that pain perception is also affected by glucose administration^30^. In the present study, it was found that glucose administration (i.p.) had a greater analgesic effect compared to fasting only while blood glucose level remained elevated (Fig. 4), suggesting that refeeding-induced analgesia is also associated with a calorie recovery. Therefore, refeeding-induced analgesia is multiple phenomena occurring simultaneously, which are clearly distinctive from simple fasting-induced analgesia.

Finally, stomach expansion might affect pain perception. Physiologically, slight distention of the stomach does not cause a significant increase in gastric pressure, but severe stomach distension may activate the affective pain system^32^. Hence it is possible that the affective component of pain from significant stomach expansion may interfere with refeeding-induced analgesia. Furthermore, PBN receives satiety signal such as ingestion, gut distention and satiety hormones directly from nucleus of the solitary tract (NTS)^33^ and is activated by not only meal-related satiety but also noxious stimuli^34^. Therefore, fullness due to stomach expansion could interfere with the analgesic effect of refeeding in formalin test by activating PBN (Figs 3c and 4d).

We have not yet determined the brain circuits that mediate refeeding-induced analgesia. Painful stimuli, such as formalin or electrical shock, is known to induce c-Fos (neural activity marker) expression in PBN^14,35^. A previous study reveals that hunger suppress inflammatory pain behavior by inhibiting PBN via the agouti-related protein (AgRP)-expressing neuron^14^. However, it is well known that the expression of c-Fos in PBN is increased by 2 h refeeding after 20–40 h acute fasting^36–38^ which is involved in the determination of meal size and meal termination during refeeding^39^. Hence, we suggest that 2 h refeeding-induced analgesia is less likely mediated by PBN.

We constructed a new model to observe spontaneous pain response in the chronic inflammatory pain model. Although clinical pain characteristics are mostly of spontaneous nature, the measurement of spontaneous ongoing pain in rodents is challenging. In our previous study, a single injection of CFA significantly increased in spontaneous paw licking and flinching behavior during only 7 days^21^. Since the motivational behavior decreases when the pain persisted for more than 7 days in the chronic pain model^40^, we tried to find out whether refeeding would have an analgesic effect even when there was a change in brain function due to chronic pain. To increase the duration of CFA-induced spontaneous pain behavior, we injected CFA twice and confirmed that CFA-induced spontaneous pain behavior persisted for 11 days in this model. Furthermore, we showed that CFA-induced spontaneous pain behavior is drastically inhibited by fasting and refeeding. This suggests that fasting and refeeding can modulate both acute and chronic spontaneous pain behavior induced by inflammation (Figs 1, 2 and Table 1).

There was no additional analgesic effect by refeeding, compared with fasting, in the CFA-induced chronic pain model (Figs 3e and 4f), while both agar refeeding and calorie recovery induced greater analgesic effect than fasting in formalin-induced acute pain model (Figs 3 and 4). In chronic pain conditions, changes in reward system related to food intake are well known^40–42^. Our study showed that the feeding pattern in the CFA-induced chronic pain model was different from that in naïve mice (Supplementary Fig. S2d,e). Therefore, changes of brain function in chronic pain model may affect the analgesic effect of refeeding. In addition, the degree of stomach expansion after 2 h refeeding seemed to be smaller than naïve mice (Supplementary Fig. S2c), suggesting that the disturbance of refeeding-induced analgesia due to stomach expansion is thought to be less in the CFA-induced chronic pain model. Indeed, the reduction in pain behavior due to normal chow refeeding was greater in the CFA-induced chronic pain model (79.99 ± 26.95%, Fig. 2c) than in the formalin-induced acute pain model (55.26 ± 18.38%, Fig. 1c). Therefore, further studies are needed to elucidate functional brain alterations related to feeding in chronic pain conditions.

Feeding and fasting drive the oscillation of energy metabolism. These oscillations are known to have positive effects not only on healthy lifespan but also on neural circuits related to cognition and emotion^43^. Thus, alternation of fasting-refeeding may have many benefits to modulate pain. Based on our findings, we propose that both fasting and refeeding modulate pathological pain more effectively through different mechanisms.

## Materials and Methods

### Animals

Male C57BL/6 mice weighing 18–25 g were purchased from DooYeol Biotech (Korea) and maintained with standard lab chow (pellet diet) and water ad *libitum* except when food was removed for deprivation experiments. All experimental procedures were reviewed and approved by the Institutional Animal Care and Use Committee (IACUC) at Seoul National University (SNU-170705-1, SNU-180518-1). All experiments were performed in accordance with relevant guidelines and regulations that were confirmed by IACUC.

### Formalin-induced pain model

20 μl of 1% formalin was injected subcutaneously into the plantar surface of the right hind paw as previously described^44^. The time mice spent licking was measured during each 5 minutes by an observer who was blinded to the treatment. Formalin-induced licking behaviors during 0–10 min after formalin injection represented the first phase and during 10–40 min after formalin injection represented the second phase. The total sum of the licking times for each phase was statistically analyzed.

### von Frey test in naïve mice

To assess mechanically evoked pain, both the 50% paw withdrawal threshold and paw withdrawal frequency was measured using von Frey filaments (North Coast Medical, Morgan Hill, CA, USA) as previously described^21^.

### Hargreaves test in naïve mice

To assess heat-evoked thermal pain, paw withdrawal latency was measured using Hargreaves method (IITC Life Science Plantar Test, Victory Blvd Woodland Hills, CA, USA) with a glass surface, heated to 30 °C. Thermal latency was measured 4 to 7 times and averaged.

### Administration of drug

Naloxone (Tocris) was diluted in 0.9% saline. SR 141716 (Tocris) was diluted in 0.9% saline with 10% DMSO and 1% tween 80. These drugs were intraperitoneally (i.p.) injected at a dose of 10 mg/kg in a volume of 10 ml/kg body weight.

### CFA (complete Freund’s adjuvant)-induced pain model

20 μl of undiluted CFA (Sigma) was injected into the plantar surface of the left hind paw. To extend the length of CFA-induced inflammatory pain, a second 20 μl of CFA injection was given 4 days after the first^40^. The CFA-induced spontaneous pain behavior was analyzed by measuring the time spent licking and flinching of the injected hind paw in the 30 min period between the hours of 08.00–10.00 as previously described^21^. To test CFA-induced mechanical allodynia, the mechanical threshold was measured using the von Frey test in the same manner as naïve mice’s paw withdrawal threshold.

### Feeding schedule for pain behavior test

In the formalin test and the von Frey test, food deprivation began between the hours of 09.00–13.00. In the CFA-induced pain model, food deprivation was performed between the hours of 09.00–10.00 on day 10 and then mice had free access to food for 2 h after 24 h of food deprivation (on day 11). For refeeding with the non-calorie agar pellet experiment, additive-free agar powder was melted in tap water (4%, in a microwave oven and then cooled in a refrigerator) and cut into about 1.5 × 1 × 0.5 cm. After 24 h food deprivation, mice had free access to 4% agar for 2 h. Water was freely accessible in all experiments.

### Measurement of body weight, blood glucose level and food intake

Glucose levels were detected in blood samples collected from the tail vein using an ACURA PLUS (automated glucometer, Korea). Body weight was measured before and after 24 h food deprivation and 2 h and 24 h of refeeding. And then, fasting and refeeding body weight was normalized to body weight before fasting. The weight of the diet was measured per cage by weighing food before and after ingestion and divided by the number of mice per cage.

### Administration of D-glucose

D-glucose was diluted in 0.9% saline and injected at a dose of 1 mg/kg (i.p.) in a volume of 10 ml/kg body weight. In the formalin test, after 15 min of D-glucose injection, formalin was injected in 24 h fasted mice. In the CFA-induced chronic pain model, on day 11, D-glucose was injected in 24 h fasted mice.

### Statistical analysis

Statistical analysis was performed using GraphPad Prism version 5.0 (GraphPad Software, USA). Comparison between two groups was made using the unpaired or paired Student’s t-test. For multiple comparisons, data were analyzed using the one-way ANOVA, repeated ANOVA or two-way ANOVA followed by the post hoc Bonferroni test. Detailed statistics for each experiment were shown in the figure legend. Data are presented as mean ± SEM. Differences with *p* < 0.05 were considered significant.

## Supplementary information

supplementary figure
