# Supplementary material for: The analgesic effect of refeeding on acute and chronic inflammatory pain
Source: Sci Rep. 2019 Nov 14;9:16873. doi: 10.1038/s41598-019-53149-7 (PMC6856519; doi:10.1038/s41598-019-53149-7)
Supplement: Supplementary file 1 — supplementary figure [file 41598_2019_53149_MOESM1_ESM.pdf]

# The analgesic effect of refeeding on acute and chronic inflammatory pain

Jeong-Yun Lee, Grace J Lee, Pa Reum Lee, Chan Hee Won, Doyun Kim, Youngnam Kang, Seog Bae Oh

## Supplementary Figures

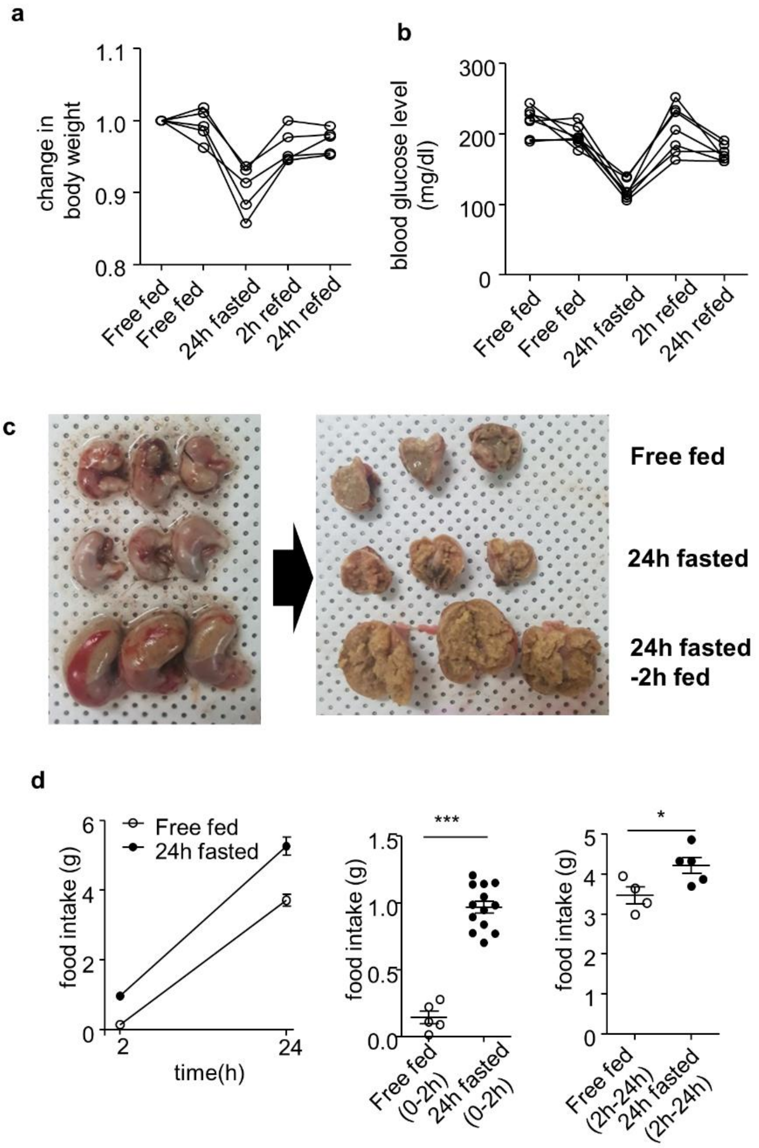


# Supplementary Figure 1

**Supplementary Fig. S1. Changes in body weight, blood glucose level and food intake by refeeding in naïve mice**

**a, b** Change in body weight and blood glucose level after refeeding in naïve mice. Refeeding for 2h after 24h fasting caused weight gain; (n=5). The decrease in blood glucose level due to fasting was also increased after 2h refeeding; (n=7). **c** Stomach expansion induced by refeeding and food inside stomach **d** Changes in food intake after refeeding. Compared with free fed group, the amount of food intake for 2h was significantly increased in 24h fasted group. The difference in food intake between two groups decreased after 2h refeeding. Data are presented as mean ± SEM. *p,0.05, ***p<0.001 (unpaired t test, two-tailed)

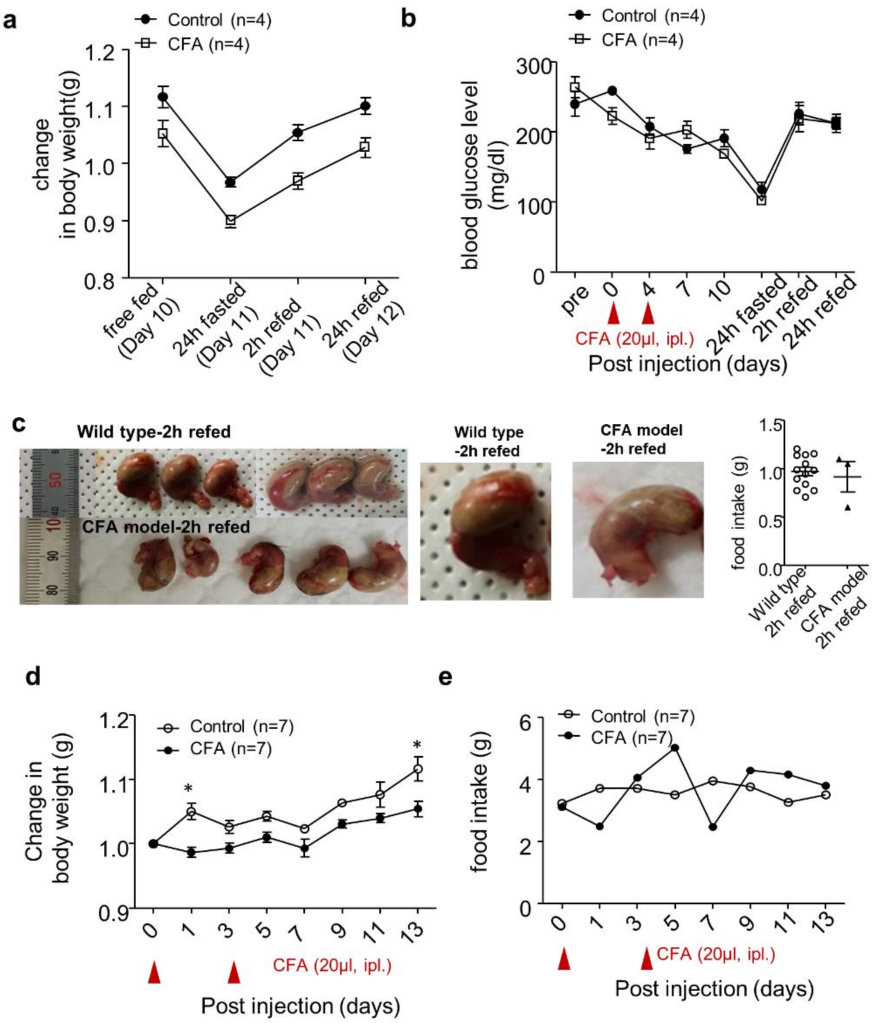


# Supplementary Figure 2

**Supplementary Fig. S2. Changes of body weight and blood glucose level by refeeding in chronic inflammatory pain model**

**a, b** Change in body weight and blood glucose level after refeeding in chronic inflammatory pain model; Control (n=4), CFA (n=4). The decrease in body weight and blood glucose level due to fasting was also increased after 2h refeeding. **c** A gross difference in stomach size after normal chow refeeding was observed in naïve in comparison to CFA induced chronic inflammatory model. The amount of food intake during refeeding period was not significantly different. **d, e** Change in food intake and body weight after CFA injection; Control (n=7), CFA (n=7). The CFA-induced chronic inflammatory pain model showed a decrease in body weight compared to the control group. Food intake was not constant. Data are presented as mean ± SEM. *$p<0.05$ (two-way ANOVAs followed by Bonferroni).

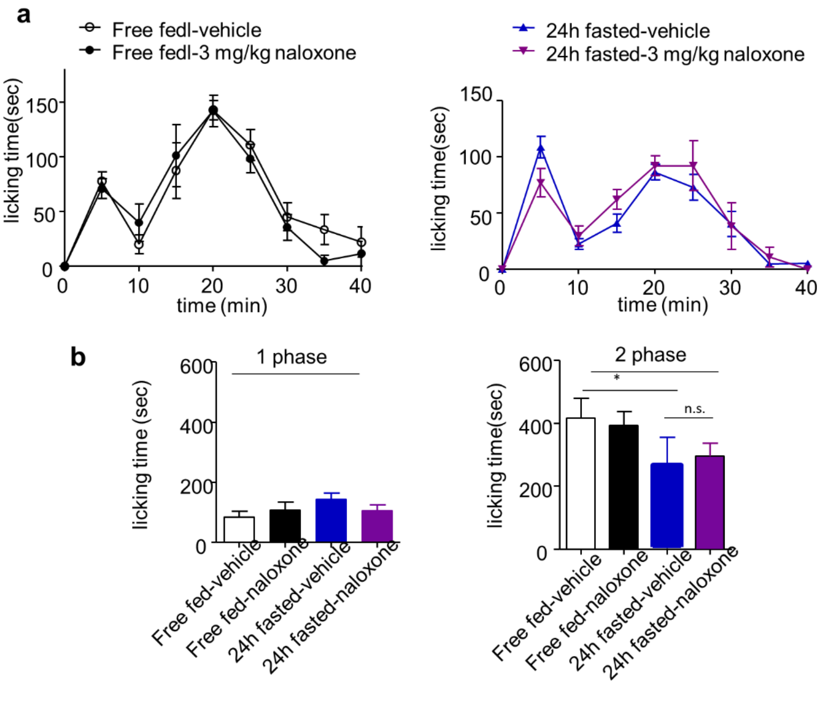


# Supplementary Figure 3

**Supplementary Fig. S3. The effect of opioid receptor antagonist on acute inflammatory pain model**

Naloxone (opioid receptor antagonist, 3 mg/kg) were intraperitoneally (i.p.) administered 30 min before the formalin injection. **a** Time course of spontaneous pain behavior following intraplantar injection of formalin; Free fed-vehicle (n=6), Free fed-naloxone (n=4), 24h fasted-vehicle (n=5), 24h fasted-naloxone (n=3) **b** Formalin-induced pain behavior was divided into two phase and the total sum of the licking times for each phase was statistically analyzed. Data are presented as mean ± SEM. *$p<0.05$ (one-way ANOVA followed by Bonferroni test)
